# Cellular and humoral immune responses against the *Plasmodium vivax* MSP-1_19_ malaria vaccine candidate in individuals living in an endemic area in north-eastern Amazon region of Brazil

**DOI:** 10.1186/1475-2875-12-326

**Published:** 2013-09-16

**Authors:** Evelyn KP Riccio, Paulo RR Totino, Lilian R Pratt-Riccio, Vitor Ennes-Vidal, Irene S Soares, Maurício Martins Rodrigues, José Maria de Souza, Cláudio Tadeu Daniel-Ribeiro, Maria de Fátima Ferreira-da-Cruz

**Affiliations:** 1 Laboratório de Pesquisas em Malária, Instituto Oswaldo Cruz, Fiocruz, Avenida Brasil 4365, Manguinhos, Rio de Janeiro, RJ, Brazil CEP: 21040-900; 2 Centro de Pesquisa, Diagnóstico e Treinamento em Malária (CPD-Mal), Fiocruz, Reference Center for Malaria in the Extra-Amazonian Region for the Secretary for Health Surveillance from the Ministry of Health, Rio de Janeiro, RJ, Brazil; 3 Laboratório de Biologia Molecular e Doenças Endêmicas, Instituto Oswaldo Cruz, Fiocruz, Rio de Janeiro, RJ, Brazil; 4 Departamento de Análises Clínicas e Toxicológicas, Universidade de São Paulo, São Paulo, Brazil; 5 Departamento de Microbiologia, Imunologia e Parasitologia, Universidade Federal de São Paulo, São Paulo, Brazil; 6 Programa de Ensaios Clínicos em Malária - Instituto Evandro Chagas, SVS, Belém, Brazil

**Keywords:** Malaria, *Plasmodium vivax*, MSP-1_19_, Cellular response, Antibodies

## Abstract

**Background:**

*Plasmodium vivax* merozoite surface protein-1 (MSP-1) is an antigen considered to be one of the leading malaria vaccine candidates. PvMSP-1 is highly immunogenic and evidences suggest that it is target for protective immunity against asexual blood stages of malaria parasites. Thus, this study aims to evaluate the acquired cellular and antibody immune responses against PvMSP-1 in individuals naturally exposed to malaria infections in a malaria-endemic area in the north-eastern Amazon region of Brazil.

**Methods:**

The study was carried out in Paragominas, Pará State, in the Brazilian Amazon. Blood samples were collected from 35 individuals with uncomplicated malaria. Peripheral blood mononuclear cells were isolated and the cellular proliferation and activation was analysed in presence of 19 kDa fragment of MSP-1 (PvMSP-1_19_) and *Plasmodium falciparum* PSS1 crude antigen. Antibodies IgE, IgM, IgG and IgG subclass and the levels of TNF, IFN-γ and IL-10 were measured by enzyme-linked immunosorbent assay.

**Results:**

The prevalence of activated CD4^+^ was greater than CD8^+^ T cells, in both *ex-vivo* and in 96 h culture in presence of PvMSP-1_19_ and PSS1 antigen. A low proliferative response against PvMSP-1_19_ and PSS1 crude antigen after 96 h culture was observed. High plasmatic levels of IFN-γ and IL-10 as well as lower TNF levels were also detected in malaria patients. However, in the 96 h supernatant culture, the dynamics of cytokine responses differed from those depicted on plasma assays; in presence of PvMSP-1_19_ stimulus, higher levels of TNF were noted in supernatant 96 h culture of malaria patient’s cells while low levels of IFN-γ and IL-10 were verified. High frequency of malaria patients presenting antibodies against PvMSP-1_19_ was evidenced, regardless class or IgG subclass.PvMSP-1_19_-induced antibodies were predominantly on non-cytophilic subclasses.

**Conclusions:**

The results presented here shows that PvMSP-1_19_ was able to induce a high cellular activation, leading to production of TNF and emphasizes the high immunogenicity of PvMSP-1_19_ in naturally exposed individuals and, therefore, its potential as a malaria vaccine candidate.

## Background

Malaria remains a serious public health problem causing high levels of morbidity and mortality in malaria-endemic regions. There were an estimated 219 million cases of malaria and 660,000 deaths in 2010 [1]. Among the five *Plasmodium* species responsible for natural infection of human, *Plasmodium vivax* has the widest geographical distribution, being the second leading cause of malaria [1]. Although usually considered a benign infection, severe *P. vivax* malaria cases have been reported worldwide [2-10]. In Brazil, *P. vivax* accounts for around 85% of clinical cases [11].

Since an effective malaria vaccine has long been envisaged as a potential tool for malaria control, two important points for its development are the identification of antigens that elicit the relevant immunological machinery and the correlation between the resulting immune system products and the clinical and/or parasitological protection induced. In this context, several antigens are being evaluated in clinical trials. To date, one candidate vaccine is currently being assessed in Phase 3 clinical trials and approximately 20 others in Phase 1 or Phase 2 trials [1]. Among these antigens, *P. vivax* merozoite surface protein-1 (PvMSP-1) is a promising candidate.

MSP-1 is the most abundant and best-studied blood-stage antigen [12]. MSP-1 is a 190–230 kDa protein present in almost all *Plasmodium* species, being synthesized in a precursor form during schizogony. Post-translational proteolytic processing of the MSP-1 precursor molecule generates different fragments (83, 28–30, 38–45 and 42 kDa). The 42 kDa fragment is processed to a 33 kDa and a 19 kDa fragments, leaving a membrane-anchored 19 kDa fragment (MSP-1_19_) on the parasite surface after its internalization in the erythrocyte [13-15].

The potential of PvMSP-1 as a vaccine candidate is based on previous studies that reported that it is highly immunogenic under natural conditions of exposure [16-24] and that it could partially protect *Saimiri* monkeys [25]. Several studies have provided evidences that MSP-1_19_ is a target for protective immunity against asexual blood stages of malaria parasites [26-28]. This protective immunity has been shown to correlate with levels of anti- MSP-1_19_ antibodies and it is also dependent of CD4 T cells [27,29,30].

Given the cumulative data supporting the potential of PvMSP-1 as a malaria vaccine, and the substantial data generated through studies in human indicating that both humoral and cellular immune responses are needed to protect against malaria, the present study aims to evaluate the acquired cellular and antibody immune responses against PvMSP-1_19_ in individuals naturally exposed to *P. vivax* or *Plasmodium falciparum* infections in a malaria-endemic area in the north-eastern Amazon region of Brazil.

## Methods

### Study setting, participants, and blood collection

The study was carried out in Paragominas (47°36′ 09.63" W, 03°12′ 11.02" S), Pará State, in the Brazilian Amazon. The samples were collected in 2004. The individuals were studied by means of a questionnaire, whereby all relevant information, including personal and epidemiological data, were collected.

Written informed consent was obtained from all volunteer donors and 10 ml of venous blood samples were drawn in *Vacutainer*® EDTA tubes (Becton Dickinson, Oxnard, CA) from 35 individuals with uncomplicated malaria at the Hospital Municipal de Paragominas (HMP). Blood samples from 17 individuals living in Paragominas but with no history of current or previous malaria episodes were included in the study as non-infected control individuals. Blood collection was performed at the day of diagnosis and patients were treated, according to the Brazilian Ministry of Health standards for malaria therapy, immediately after blood sample collection.

Thin and thick blood smears were examined for identification of malaria parasite and determination of parasitaemia by two expert malaria microscopists from HMP and from the Laboratory of Malaria Research (Fiocruz, Rio de Janeiro, Brazil) which is a reference centre in malaria diagnosis for the Brazilian Ministry of Health. Blood smears from all subjects were stained with Giemsa and examined under 1,000-fold magnification. Parasitaemia was determined by counting parasites in reference to 200 white blood cells in thick blood films, and the number of the blood parasites per millilitre was calculated.

Nonendemic control blood samples from five individuals of the laboratory staff (Rio de Janeiro, Brazil) who had neither history of malaria nor contact with a malaria transmission area, were included in the study as 'Rio controls’. The study was reviewed and approved by the Fundação Oswaldo Cruz and Instituto Evandro Chagas Ethical Committees.

### Isolation of peripheral blood mononuclear cells (PBMC)

The blood samples were centrifuged for 10 min, 400 × *g* and, after removal of plasma, the corresponding volume of RPMI-1640 (Sigma, St. Louis, Mo) medium containing 15 mM glutamin (Sigma), 10 mM Hepes (Sigma), 200 U/ml penicillin (Gibco), 200 μg/ml streptomycin (Gibco), 3 mg/ml gentamicyn (Sigma) and 2 g/L sodium bicarbonate (Sigma) was added. Subsequently, PBMC were isolated by density gradient centrifugation (Fycoll-Hypaque) and were washed twice in serum-free RPMI 1640 medium (Sigma). The cells were cryopreserved according to the method described by Ichino and Ishikawa [31]. Briefly, cells were resuspended in 4°C RPMI-1640 supplemented with 40% foetal calf serum with an equal volume of cold RPMI-1640 containing 20% dimethyl sulfoxide (Sigma) and transferred to cryotubes that were immersed in a cold ethanol bath (4°C) and placed in a -70°C freezer for at least 12 h. The samples were then transferred to a liquid nitrogen storage tank. After freezing for up to 30 days, the cells were thawed and the viability was assessed using trypan blue staining. The thawing of PBMC was performed by the immersion of cryotubes in a 37°C water-bath, followed by two washes in RPMI-1640 medium (4°C) for 10 min, 400 × *g.*

### Recovery and viability after cryopreservation

The number of viable cells was verified immediately after thawing of PBMC (*ex-vivo*) by cytometry flow using 7-aminoactinomycin D (7-AAD). This rapid and sensitive method allows the discrimination of live cells from apoptotic or necrotic cells [32]. The cells were then incubated for 20 min at 4°C with 300 μl of 10 μg/ml of 7-AAD (Sigma) in phosphate-buffered saline (PBS) containing 2% foetal calf serum (Hyclone) and 0.1% sodium azide. After incubation, 300 μl of 2% paraformaldehyde were added to the samples. Labelled samples were analysed in a FACSCalibur (Becton Dickinson and Company, Franklin Lakes, USA) and red fluorescence from 7-AAD was filtered through a 675 nm long pass filter. Approximately 10,000 events were analysed for each sample.

### *Plasmodium falciparum* crude extracts preparation

The PSS1 *P. falciparum* strain (Peixoto de Azevedo, Brazil) was cultivated *in vitro* according to the method described by Trager and Jensen [33]. The *P. falciparum* crude extract was obtained from infected erythrocytes with parasitaemia above 6%. Parasitized erythrocytes with a predominance of schizonts were washed three times with PBS. The lysis of infected erythrocytes was done by addition of 0.1% saponin and gentle shaking for 15 min. The lysates were ultrasonicated in the presence of 1 mM phenylmethylsulfonyl fluoride and centrifuged 7,000 × *g* for 15 min at 4°C in order to eliminate the cellular debris.

### Cellular proliferation assay

Cellular proliferation was analysed by using the vital staining carboxyfluorescein diacetate succinimidyl ester (CFSE, Molecular Probes). The PBMC were ressuspended in 1 ml of PBS plus 0,01% bovine serum albumin (BSA, Sigma) at 37°C. Two microlitres of a stock solution of CFSE (5 μM) were added in 1 ml of cellular suspension containing up to 1x10^6^ cells/ml. The samples were then incubated for 10 min at 37°C. Five millilitres of RPMI-1640 at 4°C were added and the samples were incubated for 5 min in ice-bath. After incubation, the cells were washed three times with RPMI-1640 and then ressuspended with 1 ml RPMI-1640 (Sigma) medium containing 15 mM glutamin (Sigma), 10 mM Hepes (Sigma), 200 U/ml penicillin (Gibco), 200 μg/ml streptomycin (Gibco), 3 mg/ml gentamicin (Sigma), 3,7 g/l sodium bicarbonate (Isofar), 100 mM piruvate (Sigma),14 mM β-mercaptoetanol (Sigma) and 10% of inactivated foetal calf serum (FCS) (complete medium). The PBMC were distributed 2.5 × 10^5^ cells/well in triplicate in 96-well flat-bottom microtiter plates (Falcon) in final volume of 200 μl of complete medium alone or in the presence of 5 μg/ml phytohaemagglutinin (PHA), 10 μg/ml *P. falciparum* crude extract or PvMSP-1_19_ (kindly provided by Dr. Mauricio Rodrigues and Dra. Irene Soares) and, then, incubated at 37°C in 5% CO_2_ for 48 or 96 h. After culture, the cellular proliferation was analysed using a FACSCalibur flow cytometer.

### Determination of cellular activation

The determination of cellular activation was done by using monoclonal antibodies against CD25, CD4, CD8 and CD21. The protocol consisted of the addition of 5-10 μl of the optimal antibody dilution to 5 × 10^5^ cells in 50 μl PBS containing 2% foetal calf serum and 0.1% sodium azide (azide PBS), followed by incubation for 20–30 min at 4°C. After two washes with PBS, the cells were resuspended in 200 μl of azide PBS. The cells were then fixed with a 2% paraformaldehyde solution and maintained in the dark until the analysis in a FACSCalibur flow cytometer. At least 10,000 events were analysed.

### Enzyme-linked immunosorbent assay (ELISA) for PvMSP-1_19_

Microtiter 96-well plates (Nunc *Maxisorp)* were coated overnight at room temperature with 50μl of 4μg/ml PvMSP-1_19_ protein in 0,05 M carbonate-bicarbonate buffer, pH 9.6. Plates were washed three times with PBS containing 0,05% Tween 20 (PBS/T20). Uncoated sites were blocked for 2 h at room temperature with 200μl of PBS/T20 containing 5% powdered-milk. After incubation, 50μl of the plasma sample diluted 1:50 in powdered-milk-containing PBS/T20 were added and the plates were incubated for 2 h at room temperature.

Plates were washed thrice with PBS/T20 and 50μl/well of mouse anti-human IgE, IgM, IgG or IgG subclass (*Sigma*) peroxidase conjugated, in optimal antibody dilution in PBS, was added. Plates were incubated for 1 h at 37°C. After washing the plates with PBS/T20, 50 μL of a solution containing 1 mg/ml of OPD (*Sigma)* and 15 μl of 30% H_2_O_2_ in citrate-phosphate buffer pH 5.0 were added. After incubation for 10–15 min at room temperature in the dark, the reaction was stopped with 50 μl/well of H_2_SO_4_. The absorbance was read at 405 nm in a spectrophotometer (Spectra Max 250; Molecular Devices, Sunnyvale, CA).

The cut-off value was determined as the mean optical density (OD) plus two standard deviations from controls that never visited malaria endemic areas. To standardize the OD data obtained in different experiments, OD index was calculated for each immunoglobulin determination as the ratio of the observed OD to the cut-off values. A sample with an OD index > 1.0 was considered positive.

### Assays for cytokine detection

The cytokine levels in plasma and supernatant cultures samples were measured by ELISA using reagents from BD Biosciences Pharmingen, USA. Briefly, 100 μl of the capture monoclonal anti-human TNF, IFN-γ or IL-10 antibodies in optimal dilutions were used to coat 96-well plates for 14 h at 4°C. After washing and blocking, 100μl of plasma samples diluted 1:2 or supernatant of the cultures were added to duplicated wells and incubated for 24 h at 4°C. After the samples were washed, biotinylated anti-human cytokine were added and the plates were incubated for 1 h at room temperature. The presence of bound antibodies was detected using streptavidin-peroxidase (Sigma Chemical Co., St Louis, USA) for 30 min at room temperature, followed by the addition of 2,2′-azinobis (3-ethylbenzthiazolinesulfonic acid) (ABTS; Sigma Chemical Co., St Louis, USA) and 30% hydrogen peroxide (Merck, Darmstadt, Germany) as the substrate. The reaction was stopped with 20% sodium dodecyl sulphate (Merck, Darmstadt, Germany), and the absorbance was read at 405 nm in a spectrophotometer (Spectra Max 250; Molecular Devices, Sunnyvale, CA). A standard curve was constructed for each cytokine by using different dilutions of human recombinant cytokines.

### Statistical analysis

For unpaired analyses, the nonparametric Mann–Whitney test was used to determine the significance of differences in plasmatic concentrations of cytokines between patients with acute malaria infection and control individuals and also among patient antibody responses. The Spearman rank correlation coefficient test was used to evaluate the correlation of epidemiological and immunological data. Student’s t-test was used to analyse differences in mean values, and chi-square analysis was applied to compare the prevalence of positive responses. Spearman rank coefficient test was also used to analyse the variable correlations. *p* values less than 0.05 were considered significant.

## Results

### Characteristics of study groups

Blood collection was performed at the day of diagnosis, before malaria treatment. Patients sought health care at Brazilian health services 6.8 ± 6.9 days after onset of symptoms. Throughout the study period, a total of 35 samples were collected from uncomplicated malaria cases (malaria group) with *P. falciparum* (n = 12) or *P. vivax* (n = 23) infection and from individuals (n = 17) living also in downtown Paragominas but with no history of current or previous malaria episodes (control group).

The malaria group consisted of 6 female and 29 men with age ranging from 12 to 67 years old (31 ± 14 years old). Twenty six percent denied prior malaria infection and 64% reported 4.7 ± 6.0 previous malaria episodes during life. The individuals from the malaria group claimed living in malaria endemic area for 19 ± 13 years. All of them presented, at time of blood sampling, symptoms and positive thick blood smears, with a parasitaemia ranging from 50 to 3,000 (1,213 ± 1,088) parasites/μl for *P. falciparum* and 75 to 5,000 (1,640 ± 1,220) parasites/μl for *P. vivax*.

Individuals from the control group claimed living in malaria endemic area for 18 ± 13 years and corresponded to 10 women and seven men with average age of 35 ± 7 years. All individuals were negative for malaria parasites as assessed by thick blood films.

### Viability of peripheral blood mononuclear cells (PBMC) after thawing

The viability of mononuclear cells after thawing was analysed and the percentages observed in malaria patients (78.1 ± 12.5%) were similar to those found in healthy individual (78.7 ± 19.6%). Likewise, no difference was observed when comparing the viability of PBMC from *P. falciparum* and *P. vivax* malaria patients.

### Phenotypic analysis of *ex-vivo* PBMC samples

The phenotypic analysis of *ex-vivo* PBMC revealed that, as expected, CD4^+^ T cells were found more frequently than CD8^+^ and CD20^+^ cells, in both malaria and control individuals (Table 1). No difference was observed when comparing the frequency of CD4^+^, CD8^+^ and CD20^+^ cells between malaria and control individuals.

**Table 1:** ***Ex-vivo *analysis of CD4**^**+**^**, CD8**^**+ **^**T cells and B cells (CD20**^**+**^**) from malaria patients and control individuals**

** Cells**	**Malaria**	**Controls**
CD4^+^	***45,6 ± 15,7***	***45,3 ± 12,4***
CD8^+^	***23,4 ± 7,9***	***24 ± 10,3***
CD20^+^	***20,1 ± 22,9***	***10,7 ± 9,6***

*(Mean ± standard deviation)*.

### Cellular activation *ex-vivo* and after 96 hours culture

The cellular activation was analysed by the expression of interleukin-2 receptor (IL-2R), using an anti-CD25 monoclonal antibody.

**Figure 1:**
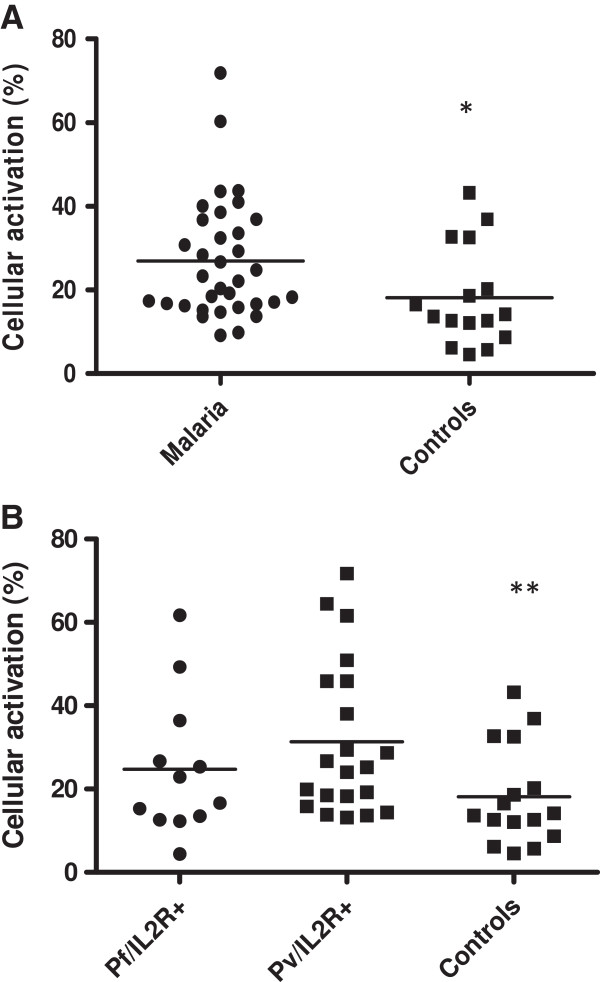
***Ex-vivo *analysis of the expression of IL-2R in PBMC by cytometry flow. (A)** PBMC from malaria patients (n = 34) and control individuals (n = 16) and **(B)** PBMC from individuals infected with *P. falciparum*, *P. vivax* and control individuals. *p = 0.01, malaria *versus* control individuals; ** *p* = 0.009 *P vivax versus* control individuals. Lines represent geometric mean.

**Figure 2:**
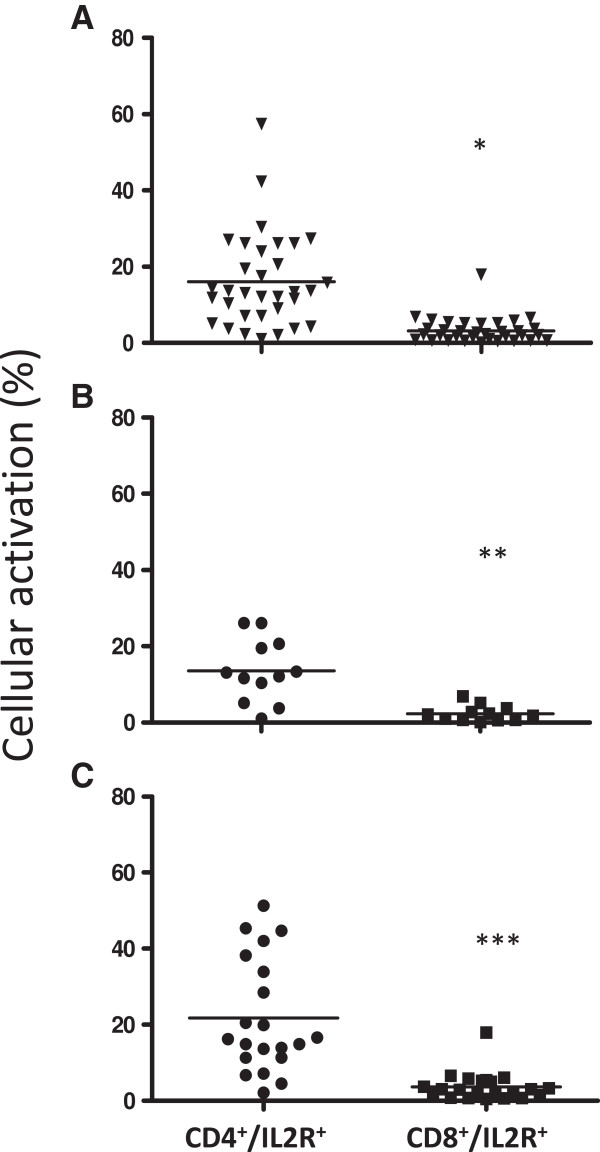
***Ex-vivo *analysis of the expression of IL-2R by cytometry flow in T cells subpopulations.** CD4^+^/IL2R + and CD8^+^/IL2R + T cells from **(A)** malaria patients (n = 34), **(B)** individuals infected with *P. falciparum* and **(C)** individuals infected with *P. vivax*. CD4^+^*versus* CD8^+^: *p < 0.0001, ** p = 0.0006, *** p < 0.0001. Lines represent geometric mean.

In *ex-vivo* analysis, PBMC from malaria individuals presented higher levels of cellular activation (27.2 ± 14.2%) than control individuals (18.2 ± 11.9%). No difference in cellular activation was observed when comparing *P. falciparum* (24.7 ± 16.8) and *P. vivax* (31.4 ± 18.3) infected individuals (Figure 1). In malaria individuals, CD4^+^ T cells were more activated (16.1 ± 12.2%) than CD8+ T cells (3.2 ± 3.3%). The same result was observed when comparing *P. vivax* (CD4^+^: 17.6 ± 13.9; CD8^+^: 3.6 ± 3.7) and *P. falciparum* (CD4^+^: 13.6 ± 8.2; CD8^+^: 2.3 ± 2) infected individuals. However, no difference was observed in the levels of cellular activation of CD4^+^ or CD8^+^ between *P. falciparum* and *P. vivax* infected individuals (Figure 2).

**Figure 3:**
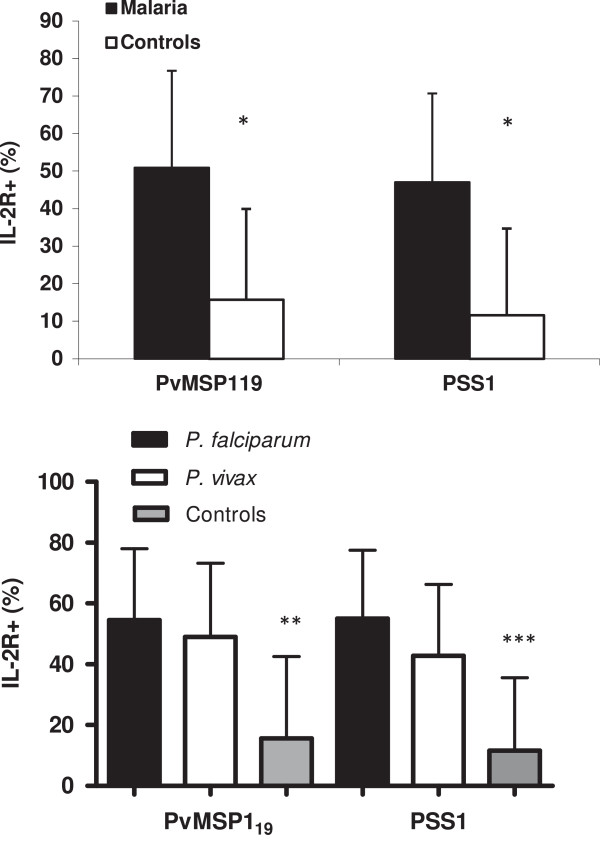
**Analysis of cellular activation by the expression of IL-2R after 96 h culture in presence of PvMSP-1**_**19 **_**and PSS1 crude antigen by cytometry flow. (A)** PBMC from malaria patients (n = 35) and control individuals (n = 17) and **(B)** PBMC from individuals infected with *P. falciparum*, *P. vivax* and control individuals. *p = 0.001 for PvMSP1_19_ and *p* = 0.0003 for PSS1, malaria *versus* control individuals; ** *p* = 0.009 for *P. falciparum versus* controls and p = 0.003 for *P. vivax versus* controls; *** p = 0.004 for *P. falciparum versus* controls and p = 0.001 for *P. vivax versus* controls.

**Figure 4:**
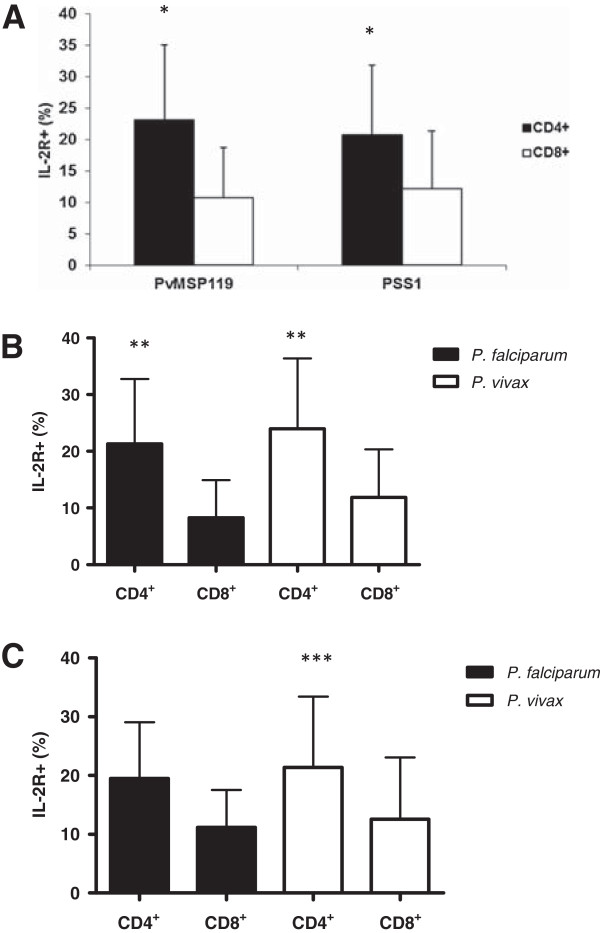
**Cytometry flow analysis of T cells activation (IL-2R**^**+**^**) after 96 h of culture in the presence of PvMSP-1**_**19 **_**and PSS1 crude antigen.** CD4^+^/IL2R + and CD8^+^/IL2R + T cells from **(A)** malaria patients (n = 35); individuals infected with *P. falciparum* and *P. vivax* in presence of **(B)** PvMSP1_19_ and **(C)** PSS1. *p = 0.0002 for PvMSP1_19_ and *p* = 0.003 for PSS1, CD4^+^*versus* CD8^+^; **p = 0.02 for *P. falciparum* and *p* = 0.003 for *P.vivax* CD4^+^ versus CD8+; ***p = 0.02 for *P. vivax*.

In 96 h culture in the presence of PvMSP-1_19_ (malaria, 50.9 ± 23.6%; controls 15.7 ± 26.8) or PSS1 crude antigen (malaria, 47.2 ± 23.4%; controls 11.6 ± 23.9%), higher levels of cellular activation were observed in malaria group than in controls individuals. The same result was observed when comparing *P. vivax* (PvMSP1_19_ 48.9 ± 24.3; PSS1 42.7 ± 23.5) and *P. falciparum* (PvMSP1_19_ 54.6 ± 23.5; PSS1 55 ± 22.6) infected individuals. However, no difference was observed in the levels of cellular activation between *P. falciparum* and *P. vivax* infected individuals (Figure 3). These activated cells were mainly CD4+ T cells (Figure 4).

### Proliferative response

**Figure 5:**
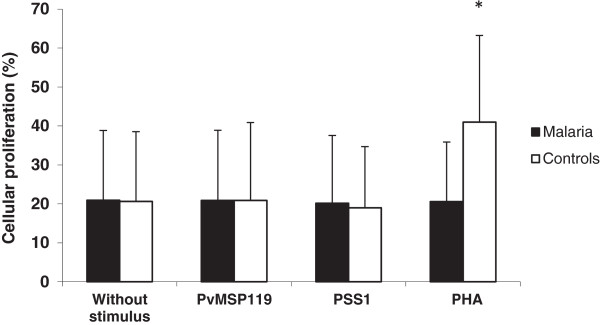
**Analysis of cellular proliferation after 96 hour culture by cytometry flow using a CFSE vital staining.** PBMC from malaria patients and control individuals in the absence or presence of plasmodial antigens (PvMSP-1_19_ and PSS1 crude antigen) or PHA mitogen. *p = 0.02 malaria *versus* control individuals

After 96 h of culture, no difference was observed when comparing different stimuli in malaria individuals. Also, in control individuals, no proliferative response was observed when cells were cultivated without stimulus or in presence of both PvMSP-1_19_ and PSS1 crude antigen. However, in presence of PHA, the proliferative response was two fold higher in control individuals than that observed in malaria patients (Figure 5).

### Cytokine profile

#### Plasmatic cytokine levels

**Figure 6:**
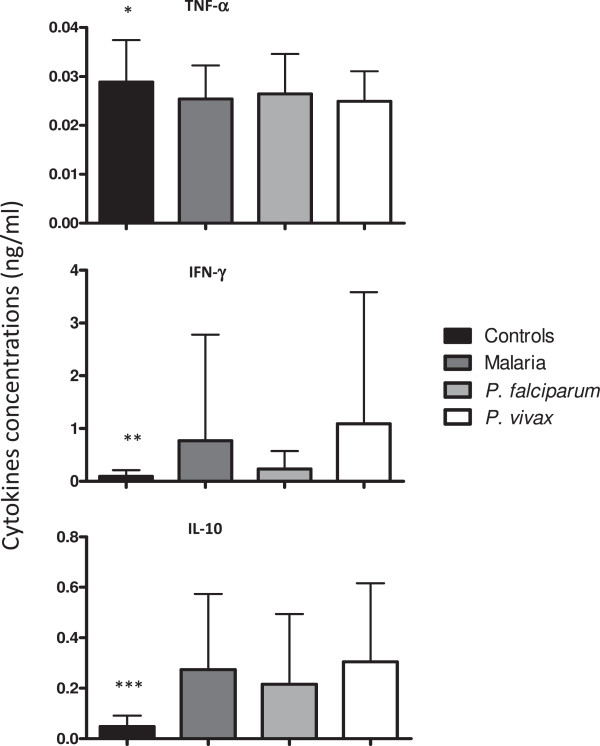
**Plasmatic concentrations of cytokines evaluated by ELISA assays.** Levels of TNF, IFN-γ and IL-10 in plasma samples from control individuals, malaria patients, individuals infected with *P. falciparum* and individuals infected with *P. vivax*. *p = 0.01 control *versus* malaria individuals and control *versus P. vivax*; ** *p* = 0.001 for controls *versus* malaria; *p* = 0.0007 control *versus P. vivax*; *** *p* < 0.0001 controls *versus* malaria and controls versus *P. vivax*; *p* = 0.001 controls *versus P. falciparum*.

**Figure 7:**
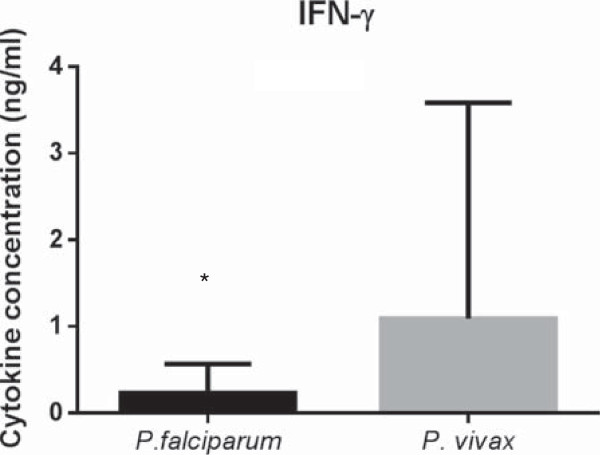
**Plasmatic concentrations of IFN-γ.** Levels of IFN-γ in plasma samples from *P. falciparum* and *P. vivax* malaria patients. *p = 0.03, *P. falciparum versus P. vivax.*

Higher plasmatic levels of IFN-γ and IL-10 as well as lower TNF levels were observed in both malaria and individuals infected with *P. vivax* than control individuals (Figure 6). No difference was observed between TNF and IL-10 levels when comparing *P. falciparum-* or *P. vivax*-infected individuals*.* However, higher levels of IFN-γ were noted in *P. vivax*- infected individuals (*p =* 0.03*)* (Figure 7).

In malaria patients, no correlation was observed between levels of plasmatic TNF or IFN and age, the number of previous malaria infection, time of residence in malaria endemic area, parasitaemia or the time elapsed between first symptoms and malaria diagnosis. However, a negative correlation was detected between plasmatic levels of IL-10 and the number of previous malaria infection (*p =* 0.0186; r = - 0.3958) in malaria individuals.

#### Cytokine levels from culture supernatant of PBMC

**Figure 8:**
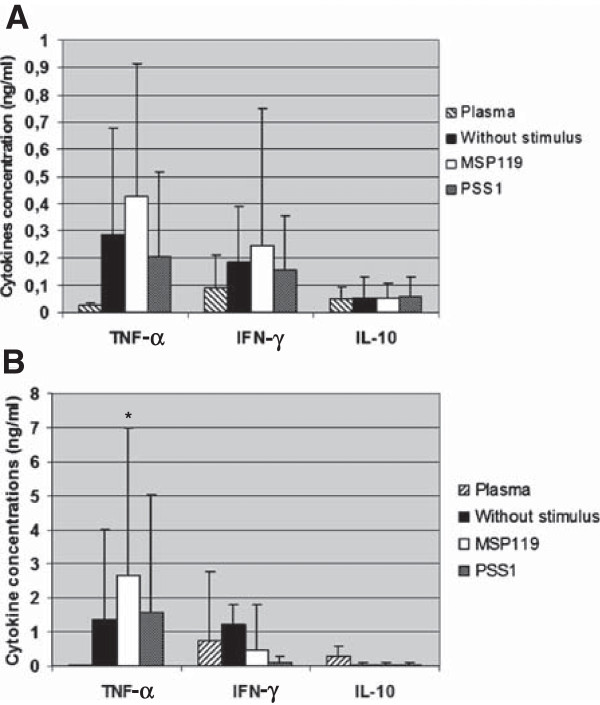
**Analysis of cytokines concentrations in plasma and in supernatant of the cultures measured by ELISA.** Levels of TNF, IFN-γ and IL-10 in plasma samples and in supernatant of the PBMC cultures from **(A)** control individuals (n = 17) and **(B)** malaria patients (n = 35). *p = 0.03, PvMSP-1_19_*versus* PSS1; *p* = 0.0006, PvMSP-1_19_*versus* without stimulus.

In the 96 h supernatant culture as represented in Figure 8, the dynamics of cytokine responses differed from those depicted in the study of the plasma samples. In presence of PvMSP-1_19_ stimulus, higher levels of TNF were observed in supernatant 96 h culture of malaria individuals’ cells when comparing with both PSS1 crude antigen (*P* = 0,03) or without stimulus (*P* = 0,0006). No statistical difference was observed between cytokines levels from supernatant cultures of *P. falciparum-* or *P. vivax-*infected patients. In malaria patients, a positive correlation was observed between the levels of TNF obtained from supernatants of cultures stimulated with PvMSP-1_19_ and the time of living in an endemic area (*P* = 0.001, r = 0.5196).

Regarding to IL-10, positive correlation was observed between the levels of this cytokine obtained from supernatants of PBMC stimulated with PvMSP1-_19_ (*P =* 0.0288, r = 0.3752) or with PSS1 (*P* = 0.033, r = 0.3661) and the time of residence in endemic area. Positive correlation was also observed between the number of previous malaria infection and the levels of IFN-γ when PBMC were stimulated with PvMSP-1_19_ (*P* = 0.0004, r = 0.5704).

### Humoral antibody response against PvMSP-1_19_

**Figure 9:**
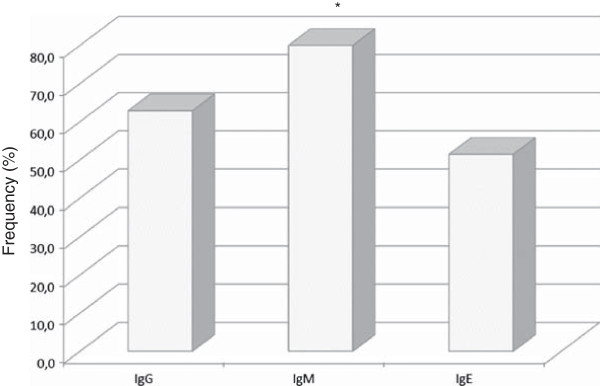
**Analysis of antibodies in sera from malaria patients.** Frequency of malaria patients (n = 35) with IgG, IgM and IgE antibodies against PvMSP-1_19_. *p = 0.02, IgM *versus* IgE.

**Figure 10:**
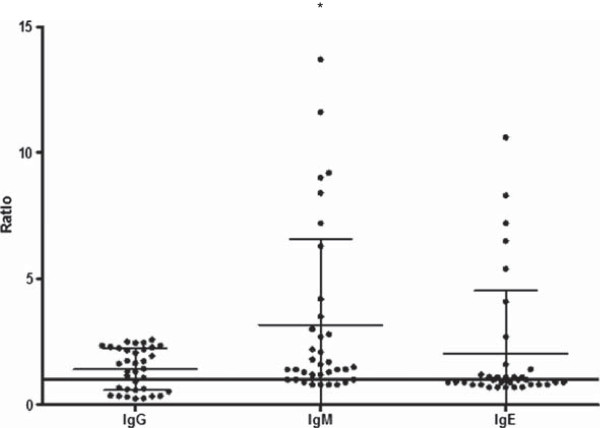
**Analysis of IgG, IgM and IgE antibodies levels against PvMSP-1**_**19 **_in malaria patients.*p = 0.005, IgM *versus* IgG. Lines represent mean with standard deviation. Continuous line represents *cut off*.

**Figure 11:**
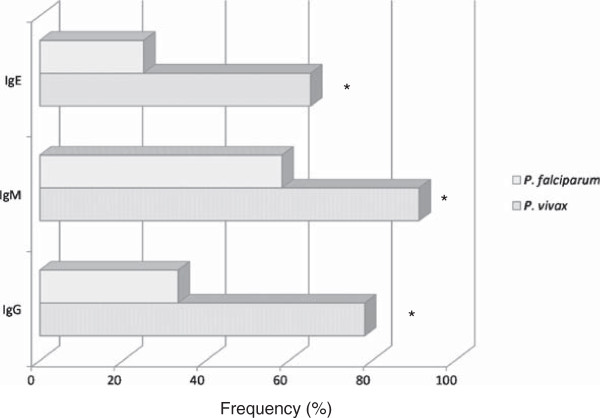
**Prevalence of antibody responses against PvMSP-1**_**19 **_**in malaria infected individuals.** Frequency of IgG, IgM and IgE antibodies in individuals with *P. vivax* or *P. falciparum* malaria. * *p* = 0.02 for IgE, *p* = 0.03 for IgM and IgG, *P. vivax versus P. falciparum*.

**Figure 12:**
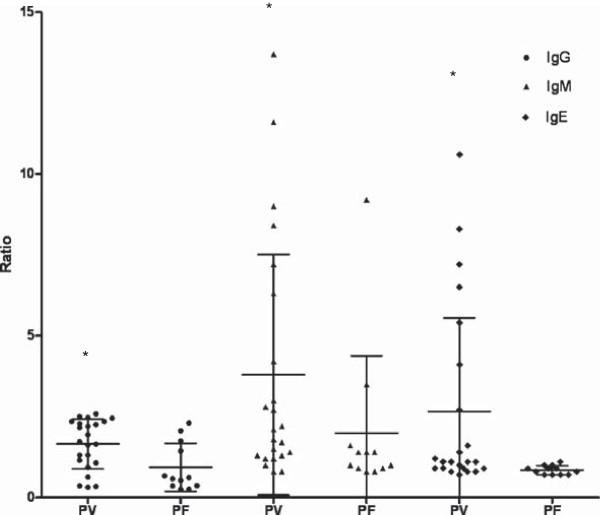
**Levels of IgG, IgM and IgE antibodies.** Analysis of antibodies levels against PvMSP-1_19_ in *P. vivax*- or *P. falciparum*- infected individuals. * *p* = 0.01 for IgG, *p* = 0.04 for IgM, *p* = 0.003 for IgE, *P. vivax versus P. falciparum*. Lines represent mean with standard deviation.

High frequency (30/35, 86%) of malaria patients presenting antibodies to IgG, IgM, IgE type and IgG isotypes against PvMSP-1_19_ was noticed. The frequencies of malaria patients presenting antibodies against PvMSP-1_19_ were 63% (22/35), 80% (28/35) and 51% (18/35) for IgG, IgM and IgE class, respectively (Figure 9). The percentage of patients presenting anti-PvMSP-1_19_ IgM antibodies was higher than the frequency of patients presenting IgE antibodies (*p* = 0.02). Besides the IgM frequency, these patients presented also higher levels of anti PvMSP-1_19_ IgM antibodies (*p* = 0.004) (Figure 10). No correlation between IgG or IgM antibodies and age, time of residence in malaria-endemic area, parasitaemia, number of previous malaria infection and time since last malaria infection was observed. However, the levels of IgE antibodies was inversely correlated with the number of previous malaria episodes (*p* = 0.01, r = -0.4061). The frequency and the levels of IgG, IgM and IgE antibodies against PvMSP-1_19_ were higher in patients infected with *P. vivax* than in patients infected with *P. falciparum* (Figures 11 and 12).

**Figure 13:**
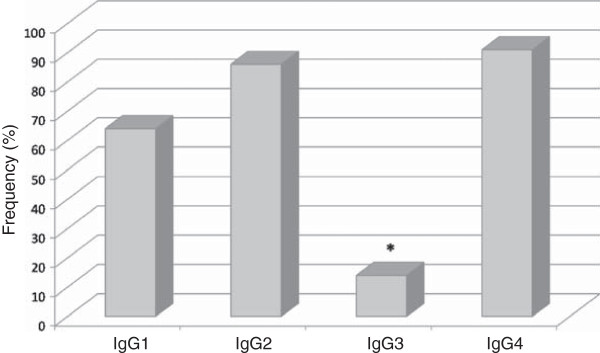
**Frequency of IgG subclasses.** Prevalence of IgG1, IgG2, IgG3 and IgG4 antibodies against PvMSP-1_19_ in IgG-positive malaria patients (n = 35). * *p* = 0.001, IgG3 *versus* IgG1; *p* < 0.0001, IgG3 *versus* IgG2 and IgG4.

**Figure 14:**
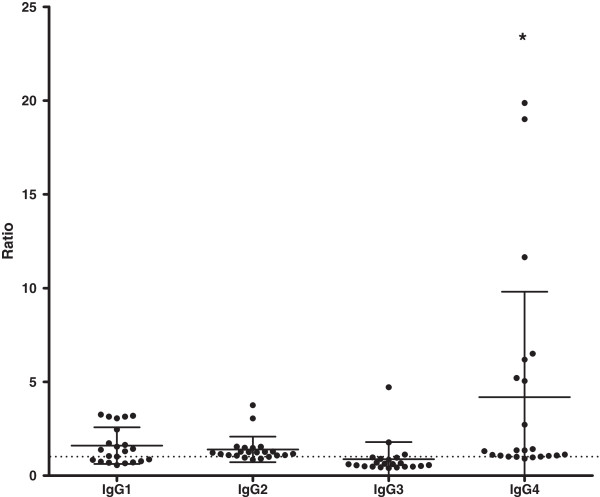
**Levels of IgG1, IgG2, IgG3 and IgG4 antibodies against PvMSP-1**_**19 **_**in malaria patients.** * *p* = 0.04, IgG4 *versus* IgG1; *p* = 0.03, IgG4 *versus* IgG2; *p* = 0.01, IgG4 *versus* IgG3. Lines represent mean with standard deviation. Dotted line represents *cut off*.

None of the 17 control individuals had detectable IgG, IgM or IgE antibodies against PvMSP1_19_ or PSS1 crude antigen. The prevalences of IgG1, IgG2, IgG3 and IgG4 subclasses in IgG-positive malaria patients were, respectively, 64% (14/22), 86% (19/22), 14% (3/22) e 91% (20/22). PvMSP-1_19_-induced antibodies were predominantly of non-cytophilic subclasses (Figure 13). Anti-PvMSP-1_19_ IgG3 antibodies were less prevalent than IgG1, IgG2 and IgG4 antibodies (*p* = 0.001 IgG3 *versus* IgG1; *p* < 0.0001 IgG3 *versus* IgG2; *p* < 0.0001 IgG3 *versus* IgG4). The levels of anti-PvMSP-1_19_ IgG4 antibodies were higher than the levels of IgG1, IgG2 and IgG3 antibodies (*p* = 0.04 IgG4 *versus* IgG1; *p* = 0.03 IgG4 *versus* IgG2; *p* = 0.01 IgG4 *versus* IgG3) (Figure 14). No correlation between IgG1, IgG2, IgG3 or IgG4 and age, time of residence in malaria-endemic area, parasitaemia, number of previous malaria infection and time elapsed after last malaria attack was observed. Also, no association was observed in the prevalence or levels of IgG1, IgG2, IgG3 and IgG4 antibodies against PvMSP-1_19_ when comparing *P. falciparum*- and *P. vivax*-malaria patients.

Positive correlations were observed between anti-PvMSP-1_19_ IgG antibodies and IL-10 levels in *P. vivax-*malaria patients (*p* = 0,0171; r = 0,7621); anti-PvMSP-1_19_ IgM antibodies and IFN-γ levels in *P. falciparum*-malaria patients (*p* = 0,0036; r = 0,7666) and; anti-PvMSP-1_19_ IgE antibodies and TNF in both *P. vivax-* and *P. falciparum*-malaria patients (*P. vivax*: *p* = 0,0432; r = 0,4081; *P. falciparum*: *p* = 0,0455; r = 0,5856).

## Discussion

In the present work, the profile of acquired cellular and antibody immune responses against PvMSP-1_19_ was evaluated in individuals naturally exposed to *P. vivax* and *P. falciparum* infections in a malaria-endemic area in the north-western Amazon region of Brazil.

It is well known that suboptimal cryopreservation could result in a significant decrease of cell number and viability, causing alterations on the cellular phenotype and on the immune response to specific antigens. To avoid troublesome events, a protocol to successfully recovery PBMC after freezing and thawing was previously standardized [34]. Thus, in the present study, an important frequency of mononuclear cell viability after thawing was observed (around 78%), regardless the individuals being infected or not. Because 75% PBMC viability is required for lymphocyte proliferation assays, representing the ability of cells to respond to the antigenic or mitogenic stimulation, independently of the nature or intensity of the stimulus [35], the cryopreservation procedure seems not have been a limiting factor in the present study.

The phenotypic analysis of *ex-vivo* PBMC revealed that CD4^+^ T cells were found more frequently than CD8^+^ and CD20^+^ cells, in both malaria and control individuals. In malaria individuals, a higher prevalence of activated CD4^+^ than CD8^+^ T cells was observed, in both *ex-vivo* and in 96 h culture in presence of PvMSP-1_19_ and PSS1 antigen. It can be speculated that CD4^+^ T cells were the most stimulated cells due the nature of antigenic stimulus because CD4^+^ T cells are activated by the parasite in blood stage, while CD8^+^ T cells are most often activated during the hepatic stage of parasite life cycle [36].

Low proliferative responses in the presence of MSP-1_19_ of *P. falciparum* and *Plasmodium chabaudi chabaudi* have already been demonstrated [37,38]. The results also showed a low proliferative response against PSS1 crude antigen or PvMSP-1_19_ after 96 h culture. This low proliferative response may occur because the majority of activated cells undergo activated-induced cell death, an active cell suicide mechanism of widespread biological importance that constitutes the physiological response of normal cells to activation and is believed to control the number of antigen-stimulated cells during the immune response [39].

Anti-inflammatory cytokines are involved in a feedback mechanism to regulate the expression of pro-inflammatory cytokines, and prevent the pathological effects that may result from their continuous secretion. Previous *in vitro* studies have shown that IL-10 suppresses the expression of malaria parasite-induced production of TNF by PBMC [40]. In fact, in the present study, high plasmatic levels of IL-10 and low plasmatic levels of TNF were observed in malaria patients.

Parasite clearance seems to be related to IL-10 and IFN-γ levels. Here, an intense secretion of IFN-γ and IL-10 as detected by dosage of plasmatic levels, were shown in malaria patients. However no correlation was found between parasitaemia and IFN-γ or IL-10 levels. The high levels of IL-10 and IFN-γ are in agreement with previous clinical reports that IL-10 is up-regulated in concern with IFN-γ [41,42], suggesting that IL-10 may be up-regulated as a direct consequence of IFN-γ production as part of homeostatic feedback mechanism to limit IFN-γ-mediated pathology, as is seen in murine malaria infections [43].

IL-10 levels were similar in *P. vivax* and *P. falciparum* infected individuals. However, besides plasmodial species, other factors, like the number of previous malaria infections, may influence the levels of this and other cytokines. In fact, a negative correlation between number of previous malaria infections and IL-10 levels was found. In addition, previous studies have shown that an adaptive type 1 regulatory CD4^+^ cells have been identified as the main source of IL-10 in experimental murine infection with *Plasmodium yoelii*[44]*,* while in *P. chabaudi* infections in mice, an activated effector TH_1_ cells were major IL-10 producers [45]. Understanding how these regulatory cells are induced could help to explain differences in IL-10 production in human malaria.

High production of TNF in malaria individuals is related to the development of severe malaria. In the present work, lower than expected plasmatic TNF levels (lower than those observed in control individuals) were detected in malaria patients. Interestingly, PvMSP-1_19_-stimulated PBMCs from malaria patients showed higher TNF response than those recorded in both PSS1-stimulated and non-stimulated PBMCs. The production of TNF in recall to PvMSP-1_19_ may suggest a protective role of PvMSP-1_19_ immune response because TNF has been related to parasite clearance.

A great majority of studied individuals presented antibodies against PvMSP-1_19_, independently of the reported number of previous malaria attacks or the time of residence in a malaria-endemic area, indicating that this protein is immunogenic in natural conditions of exposure and seems to be independent of the time of exposure. The high immunogenicity of PvMSP-1_19_ may be reflex of its limited polymorphism, because independent groups demonstrated that PvMSP-1_19_ is conserved [17,46,47]. Another possible explanation is that PvMSP-1_19_ is the single fragment that remains on the parasite surface during red cell invasion through glycosylphosphatidylinositol (GPI) anchor [13] and studies have described GPI as a potent agonists of toll-like receptors that may provide the adjuvant required for stronger immune responses [48].

Anti-PvMSP-1_19_ antibodies were not detected in the sera of five individuals from malaria group (one with vivax and four with falciparum malaria). The absence of anti-PvMSP-1_19_ antibodies could be related to immunosuppression observed in humans in malaria infection [49,50], however, this hypothesis seems unlikely because these non-responders individuals presented antibodies against PSS1 crude antigen. Another possible explanation for the absence of these antibodies is that PvMSP-1_19_-specific B cells were present, although circulating antibodies titers were undetectable. Alternatively, the lack of PvMSP-1_19_ antibody response may be due the genetic restriction of immune response that has been described to several plasmodial recombinant proteins and synthetic peptides [24,51-55]. However, in a recent study performed in the Brazilian Amazon region, no association between HLADRB1* and HLADQB1* allelic groups and the antibody response against PvMSP-1_19_ was found [24].

Considering that different *Plasmodium* antigens in the same population as well as the same *Plasmodium* antigen in different populations can induce different antibody profiles, it was also evaluated the frequency and levels of IgG, IgM and IgE class and IgG subclass against PvMSP-1_19_. IgM antibodies were the most prevalent and the one with the highest levels. PvMSP-1_19_-induced IgG antibodies were predominantly of non-cytophilic subclasses. Different data have been reported for individuals living in other Brazilian-endemic areas with different levels of exposure where the PvMSP-1_19_-induced antibodies were predominantly of IgG1 subclass [20,23]. Differences in antibody profile may be due to transmission intensity. In fact, previous study has demonstrated that the levels of IgG1 and IgG3 specific antibodies were low among individuals with long-term exposure (~19 years) when compared to subjects less and sporadically exposed (< 1 year) [20]. In this concern, one must emphasize that the individuals from malaria group claimed living in malaria endemic area for 19 years. Another additional hypothesis could be related to cytokine modulation of specific antibodies production because IL-10 has been reported to demonstrate both potentiating and inhibiting IgE and increasing IgG4 productions [56] as well as to be associated with IgM antibodies against *P. vivax*[57]. In addition, associations between polymorphism in cytokine genes and anti-plasmodial antibody response have been reported [53]. One other possibility could be the context of the response because co-infection with helminths is known to shift the Th_1_ to the Th_2_ pattern of immune response modulating the IgG subclass expression. Independently of its origin, the differences between antibody profile reported in the present study and the aforementioned underline the importance of conducting immunoepidemiological studies in different malaria endemic areas where transmission intensities and human genetic background are different.

Associations between antibody responses with time of residence and/or number of previous episodes have been commonly reported to several malaria antigens [52,58,59]. However, in the present study, no association between levels of PvMSP-1_19_ antibodies and number of previous malaria episodes was observed. This finding may reflect that people living for a longer period of time in the region may have acquired some degree of clinical immunity after experiencing a number of infections, therefore, reporting less episodes of clinical malaria in the more recent years. Interestingly, only the levels of IgE antibodies were inversely correlated with the number of previous malaria episodes. Thus, it can be supposed that higher levels of IgE antibodies may have an important role against clinical malaria. Similar results were already reported that shown that high levels of malaria specific IgE were associated with reduced risk for subsequent clinical malaria episodes [60].

In this study the frequency and the levels of IgG, IgM and IgE antibodies against PvMSP-1_19_ were higher in individuals infected with *P. vivax* than *P. falciparum* or who had reported the last malaria episode due *P. vivax. S*imilar results were reported elsewhere where sera from subjects who had had *P. vivax* in their last malaria clinical episode presented higher levels of antibodies when compared with those whose last malaria episode was due to *P. falciparum*[20].

The individuals infected with *P. falciparum* studied in this work reported previous infection with *P. vivax*. This may explain the recognition of PvMSP1_19_ antigen by antibodies from individuals infected with *P. falciparum*. However, the similarity between PfMSP1 and PvMSP1 antigens may also explain, at least in part, the recognition of PvMSP1 by patients infected with *P. falciparum*, although a recent study has shown that sera from immunized mice with PfMSP1_19_ or PvMSP1_19_ failed to cross-react with heterologous antigen [61].

In conclusion, the results presented here shows that PvMSP-1_19_ was able to induce a high cellular activation leading to production of TNF, emphasizing the high immunogenicity of PvMSP-1_19_ in naturally exposed individuals and therefore its potential as a malaria vaccine candidate.

## Competing interests

The authors declare that they have no competing interests.

## Authors’ contributions

EKPR designed the study, carried out the experiments, performed the statistical analysis and drafted the manuscript; PRRT, VEV and LRPR participated in its design, carried out the experiments and reviewed the manuscript; MMR and ISS produced and provided the PvMSP-1_19;_ JMS enabled and facilitated the performance of experiments in Paragominas; CTDR reviewed the manuscript and MFFC conceived the study, participated in its design and coordination and reviewed the manuscript. All authors have read and approved the final manuscript.
